# Autonomic Nervous System, Cognition, and Emotional Valence During Different Phases of the Menstrual Cycle—A Narrative Review

**DOI:** 10.3390/neurosci6030078

**Published:** 2025-08-13

**Authors:** Sankanika Roy, Elettra Agordati, Thomas D. W. Wilcockson

**Affiliations:** 1 Department of Neurology, University Hospitals of Leicester, Leicester LE5 4PW, UK; 2 Department of Renal Medicine, Nottingham University Hospitals, Nottingham NG5 1PB, UK; elettra_la_ma@hotmail.it; 3 School of Sport, Exercise and Health Sciences, Loughborough University, Loughborough LE11 3TU, UK; t.wilcockson@lboro.ac.uk

**Keywords:** autonomic nervous system, menstrual cycle, cognition, emotional valence, biological women

## Abstract

The menstrual cycle affects the autonomic nervous system (ANS), cognition, and emotional valence in all biological women. There exists a complex relationship between hormonal fluctuations, ANS, cognition, and emotional valence during the different phases of the menstrual cycle, which includes menstruation, the follicular phase, ovulation, and the luteal phase. Hence, this narrative review is an attempt to comprehensively understand the effects of the menstrual cycle on the structural and functional integrity of the ANS. In order to provide a comprehensive understanding of the complex relationship between the menstrual cycle, hormonal fluctuations, and ANS function in biological women, this review examines key parameters, including heart rate variability (HRV), baroreflex sensitivity (BRS), muscle sympathetic nerve activity (MSNA), and pupillary light reflex (PLR), to investigate how these physiological systems are dynamically influenced by the cyclical changes in hormone levels and how these fluctuations impact various physiological and psychological outcomes, such as mood, cognition, and emotional regulation. There have been several studies previously performed to assess these parameters during different phases of the menstrual cycle. However, the results have been contradictory; therefore, this review explores possible reasons behind these inconsistent results, with likely reasons including irregularity in the menstrual cycles and differences in hormonal fluctuations between different women during similar phases of the menstrual cycle. Overall, there appears to be evidence to suggest that the menstrual cycle has both direct and indirect effects on ANS, cognition, and emotional valence, whilst measures of ANS may provide a means for assessing the effect of the menstrual cycle.

## 1. Introduction

The autonomic nervous system (ANS) plays a fundamental role in regulating various homeostatic mechanisms of the human body, including cardiovascular regulation, neuroendocrine responses, pain perception, and cognitive and behavioural processes [1]. Its relationship with the brain involves the constant integration of sensory information from both internal and external environments, including viscero-sensory, humoral, hormonal, and psychological stimuli, which allows for adaptive responses to maintain homeostasis [2]. While gender differences in ANS regulation are well-documented [3], the impact of the menstrual cycle on the ANS function of biological women remains an area of significant interest. This review explores the impact of the menstrual cycle on the structural and functional integrity of the ANS in biological women, i.e., how well the ANS is functioning in terms of physical soundness and efficiency. The cyclical fluctuations of oestrogen and progesterone [4] exert direct and indirect effects on the ANS, with direct effects involving the hormonal modulation of autonomic pathways, while indirect effects arise from the influence of these hormones on emotional valence and cognitive function [5,6].

Understanding these complicated relationships is crucial for several reasons. Firstly, it offers valuable insights into the physiological underpinnings of conditions like premenstrual syndrome (PMS) and premenstrual dysphoric disorder (PMDD), which affect a significant proportion of biological women—ranging from 40 to 90% for PMS and from 1 to 10% for PMDD [7,8]. These conditions are characterised by symptoms including mood swings, anxiety, irritability, fatigue, and cognitive difficulties, which are often linked to ANS dysregulation [9]. Secondly, this knowledge can inform personalised healthcare approaches for women. By recognising the cyclical nature of ANS function, clinicians can tailor interventions, such as exercise routines or medication schedules, to optimise the health and well-being of biological women across the menstrual cycle. Thirdly, this research has the potential to enhance our understanding of a biological woman’s performance in various domains, including sports and athletics, during different phases of the menstrual cycle. By identifying how the menstrual cycle influences ANS function, we can develop strategies to optimise training and performance during these different phases.

To comprehensively assess the impact of the menstrual cycle on the ANS, this review will examine key parameters, including heart rate variability (HRV), baroreflex sensitivity (BRS), muscle sympathetic nerve activity (MSNA), and pupil light reflex (PLR). HRV, BRS, and PLR offer distinct, yet complementary, perspectives on how the menstrual cycle influences ANS function. HRV measures the fluctuations in the heart rate (HR), which primarily gauges the balance between sympathetic and parasympathetic activity within the cardiovascular system. BRS measures the body’s ability to sense fluctuations in blood pressure (BP) via baroreceptors and helps modify BP through changes in HR and vascular tone, which provides insights into the dynamic relationship between these branches of the ANS in regulating BP. In contrast, PLR, which measures the constriction and dilation of the pupil in response to light, primarily provides an insight into the non-cardiovascular regulatory functions of the ANS. While some overlap exists between these measures, each provides unique insights into ANS function. With HRV and BRS offering a broad assessment of autonomic balance with respect to cardiovascular homeostasis, PLR focuses on the antagonistic actions of the iris sphincter and dilator muscles, assessing the ANS function and providing information regarding higher cortical functions including emotional valence and cognition. By investigating these parameters across different phases of the menstrual cycle, this review aims to examine how the ANS is affected by the menstrual cycle and how this has an impact on the various physiological and psychological outcomes in biological women.

While these measures provide valuable information and are the focus of this review, other methods exist for assessing the effects of the menstrual cycle on the ANS, such as microneurography (for a direct assessment of muscle sympathetic nerve activity (MSNA)), salivary biomarkers (reflecting neuroendocrine activity), and electrodermal activity (e.g., skin conductance) as an index of sympathetic arousal, which can provide more nuanced insights into the menstrual cycle and the ANS. However, it is important to acknowledge the limitations of some of these measures. Microneurography is an invasive technique that may not be suitable for all participants, and salivary biomarker and electrodermal activity measurements can be influenced by various factors, such as stress, temperature, and hydration. HRV, BRS, and PLR therefore offer several advantages as they provide objective, physiological measures of ANS function and they can be measured relatively easily and non-invasively, making them suitable for longitudinal studies and real-world applications.

Overall, this review examines the multifaceted impact of the menstrual cycle on the ANS in biological women. By employing a combination of HRV, BRS, PLR, MSNA, and other relevant assessments, we can explore the physiological underpinnings of conditions like PMS and PMDD, providing insights which could lead to personalised healthcare approaches and optimise women’s health and well-being throughout their menstrual cycle.

## 2. The Menstrual Cycle and Hormonal Changes

**Figure 1:**
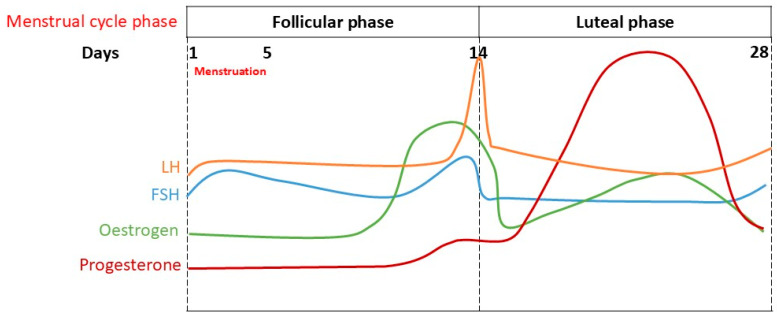
Schematic representation of fluctuations in the hormones oestrogen, progesterone, luteinising hormone (LH), and follicle-stimulating hormone (FSH) during the different phases of the menstrual cycle.

Given the significant impact of the menstrual cycle on physiological processes, including the regulation of the ANS, understanding these mechanisms is crucial for optimising women’s health. This cycle is divided into the follicular phase, ovulation, the luteal phase, and menstruation. Sex hormones, oestrogen, and progesterone fluctuate during the different phases of the menstrual cycle. During the late follicular phase, which is from day 10 of menstruation to day 14 of the 28-day menstrual cycle, there is a rapid rise in the level of oestrogen. The follicular phase can be typically divided into early and late phases. The early follicular phase lasts from day 1 of menstruation to around days 10–12 of the cycle; during this period, progesterone remains low. However, oestrogen levels gradually start to rise. The late follicular phase spans from around day 10–12 to ovulation; during this phase, oestrogen levels become very high and reach their peak. It is important to note that the duration and hormonal profiles of these phases vary amongst women and may not always present analogously. Ovulation occurs at the end of the follicular phase after oestrogen reaches its peak, resulting in a luteinising hormone (LH) surge, which is responsible for ovulation. This phase is followed by a fall in the level of oestrogen in the luteal phase. During the luteal phase, progesterone is the dominant hormone. In the mid-luteal phase (6 or 7 days before menstruation), progesterone is at its highest level, while in the late luteal phase, there is a reduction in the levels of both oestrogen and progesterone, followed by menstruation [4], thus completing one cycle (please refer to Figure 1). Table 1 contains a summary of the key hormonal changes and physiological and psychological characteristics at each stage of the menstrual cycle.

## 3. Menstrual Cycle and Psychopathology

### 3.1. Emotional Valence

It is well understood that the phases of the menstrual cycle cause fluctuations in different hormones, and these fluctuations can also affect emotional valence and behaviours. Oestrogen receptors (ERα and ERβ) mediate the effects of oestrogen on neurotransmitters and neuronal networks, both of which are important for the development of empathy. ERα has been attributed to empathy enhancement; on the other hand, ERβ has been shown to suppress empathy [10]. The balance between these two receptor responses, as well as the availability of receptors in crucial areas of the brain, e.g., the amygdala and prefrontal cortex, determines homeostasis. Fluctuating levels of oestrogen during the menstrual cycle could have a positive or negative influence on empathetic response. Phases with low oestrogen levels have been associated with low mood in healthy biological women; in patients with PMDD, the association is linked to depression [11]. Oestrogen affects dopamine (DA) metabolism; it can increase levels of DA in the striatum, hypothalamus, and anterior pituitary, thus possibly playing a role in reducing depression. Long-term treatment with oestrogen increases the density of DA receptors in striatum and nucleus accumbens [12]. Oestrogen is a sex steroid which has anti-inflammatory effects related to depression; it reduces astrocyte- and microglia-induced inflammatory responses in the brain and decreases the release of inflammatory factors such as IL-6, TNF-α, and NO [13]. Hence, menstrual phases associated with low oestrogen levels could be associated with low mood, depression, and affect disorders. Therefore, these findings suggest that the fluctuating hormonal levels of the menstrual cycle can significantly impact emotional well-being, particularly in conditions like PMDD, and have important implications for the development of effective treatment strategies.

### 3.2. Cognitive Functioning

Similarly, cognitive function is thought to be influenced by the menstrual cycle, with both progesterone and oestrogen being shown to have significant effects on cognition. Oestrogen and progesterone receptors have been found in several areas of the brain that are associated with cognitive functioning [14,15]. Both hormones can cross the blood–brain barrier and are useful for synaptic formation and neuroprotection [14,15]. Oestrogen has been shown to have effects on memory tasks [16], while oestrogen depletion due to menopause is associated with cognitive decline [17]. Oestrogen interacts with the cholinergic system, which can modulate attention, learning, and memory; oestrogen can also reduce anticholinergic-induced verbal memory impairment [18]. Progesterone is considered to have neuroprotective effects, especially for maintaining cognitive function [14]. A recent review found inconsistent findings regarding cognitive functioning during the different phases of the menstrual cycle; most studies investigated oestrogen and its association with cognition. On the other hand, the effect of progesterone remains unexplored [19]. Research on the menstrual cycle, cognition, and hormonal fluctuations is still in its early stages; further research is required to better understand their relationship and could lead to opportunities for developing therapeutic management for biological women suffering from cognitive dysfunction associated with the menstrual cycle. However, if the menstrual cycle is affecting cognitive function, then there are broad implications for recognising and addressing potential cognitive fluctuations in women and potentially being aware of periods of peak and sub-optimal cognition.

## 4. Effects of the Menstrual Cycle on the Autonomic Nervous System

The menstrual cycle can have a direct effect on psychopathology in terms of emotion and cognition. However, the underlying mechanisms through which the menstrual cycle brings about physical symptoms are likely via alterations in the activity of the ANS. Fluctuations in hormone levels across the menstrual cycle can significantly impact the balance between sympathetic and parasympathetic activity, leading to variations in HR, BP, and other physiological responses that can contribute to emotional and cognitive changes. There are numerous ways of assessing the ANS, with this review focusing on HRV, BRS, MSNA, and PLR.

### 4.1. Assessment of the Autonomic Nervous System

#### 4.1.1. Heart Rate Variability

Heart rate variability (HRV) is used for a non-invasive assessment of cardiac autonomic function. HRV can be assessed by time or frequency-domain measures. From a continuous ECG, QRS complexes are identified and the normal–normal (NN) intervals (all intervals between adjacent QRS complexes) are determined. Time-domain variables include the mean NN interval, the mean heart rate standard deviation of the NN interval (SDNN), RMSSD, the square root of the mean squared differences in successive NN intervals, and NN50, which is the number of interval differences in successive NN intervals greater than 50 ms. RMSSD can be considered as one of the most efficient measures of vagal tone [20,21]. Frequency-domain parameters are obtained from short-term recordings as very-low-frequency (VLF), low-frequency (LF) and high-frequency (HF) spectral components. HF components (0.15–0.40 Hz) are believed to arise from vagal control; for the LF components (0.04–0.15 Hz), however, there is some debate, with some evidence suggesting LF components are associated with sympathetic nerve system (SNS) activity only [22,23], while other evidence suggests a concept involving both SNS and parasympathetic nervous system (PNS) involvement [24]. HRV determines cardiac autonomic control and is utilised for the prognostication of cardiovascular diseases. Reduced HRV is associated with an increased risk of mortality and morbidity [25,26]. The utilisation of HRV is not limited to cardiovascular diseases; it has its applications in neuroscience and has been shown to predict psychological outcomes [27]. More specifically, the vagally mediated component of HRV (vmHRV) has recently been studied extensively and has emerged as a biomarker for both physical and mental health [28,29,30].

#### 4.1.2. Baroreflex Sensitivity

Baroreflex is the mechanism by which ANS maintains cardiovascular homeostasis. Baroreceptors are located at the carotid bifurcation and the aortic arch. They are stretch receptors that are activated by sudden changes in BP, and are responsible for sending information to the central nervous system (CNS)—especially the cardiovascular regulatory centre in the medulla oblongata, which brings about rapid changes in the HR and peripheral vasculature via the SNS and PNS to stabilise the BP. BRS is a measure of baroreflex function and is defined as the change in R-R intervals (of an ECG recording) in milliseconds (ms) duration to each mmHg of BP change, with a normal value of ~15 ms/mmHg [31]. BRS can also be illustrated by determining the slope of the relationship between mean arterial pressure and HR. BRS is currently an established tool for the assessment of the functions of the ANS with respect to the regulation of the cardiovascular system. It also provides valuable information in the clinical setting.

#### 4.1.3. Muscle Sympathetic Nerve Activity

Muscle sympathetic nerve activity describes the electrical activity of sympathetic nerves innervating the skeletal muscle vasculature and regulating peripheral vascular resistance in order to control systemic BP [32]. Nerve activity can be directly recorded by a technique called microneurography via introducing a recording electrode into an accessible nerve. MSNA is an efficient assessor of the BRS as it is under the strict modulation of the baroreflex. Any external/internal stimuli that change BP can significantly increase or decrease sympathetic nerve activity and modulate systemic BP [33].

#### 4.1.4. Pupil Light Reflex

The PLR involves pupillary constriction and subsequent dilation in response to light [34]. Pupillary constriction is mediated by the PNS, and dilation is mediated by the SNS. PLR is essential for optimising visual acuity under varying levels of luminance [35] but also offers valuable insights into the functioning of the ANS [36]. As a process influenced by both autonomic and cognitive factors, the PLR serves as a valuable biomarker for assessing ANS and cognitive function [37]. The PLR occurs in response to light stimulation. Light activates photoreceptors in the retina, which then initiate the reflex leading to pupillary constriction by sending signals through the optic nerve to the pretectal area of the midbrain, resulting in the activation of the parasympathetic efferent fibres projecting to the sphincter pupillae muscle of the iris, subsequently resulting in pupillary constriction [38]. Pupillary dilation is mediated by the SNS; it is a three-order neuron pathway that supplies the dilator pupillae muscle, which causes pupillary dilation [39].

However, the PLR is not solely a reflexive response. Cognitive factors, such as attention, arousal, and task demands, can influence the magnitude and speed of pupillary constriction [40]. For example, faster PLR responses are associated with an increased cognitive load [41]. The influence of cognitive factors on the PLR is thought to involve interactions between multiple brain regions. The prefrontal cortex, which is involved in attention, decision-making, and executive control, plays a role in modulating the PLR [42]. The authors of [42] have demonstrated that the prefrontal cortex (specifically the frontal eye field) is involved in the selective perceptual and attentional modulations of PLR. Furthermore, focusing attention increases PLRs to probes in attended locations and suppresses PLRs to probes in other locations [43]. Therefore, when attention is focused on a specific task, the prefrontal cortex may suppress the PLR to allow for better visual processing. The anterior cingulate cortex, which is involved in emotional processing and conflict monitoring, also influences the PLR [44]. Emotional arousal, which is mediated by the anterior cingulate cortex, influences the PLR such that pupillometry itself can be used as a measure of autonomic arousal states within the anterior cingulate cortex [44]. The anterior cingulate cortex modulates the parasympathetic response that is responsible for pupil constriction [45]. The thalamus, which is a junction for sensory information including visual input, interacts with these regions to modulate the PLR. The thalamus plays a key role in regulating arousal levels, and changes in arousal can affect pupil dynamics [46]. Additionally, the brainstem, which contains nuclei that control the ANS including the parasympathetic response responsible for the PLR, is involved in regulating the reflex [47]. The locus coeruleus (LC) plays a crucial role in modulating the PLR by regulating arousal, attention, and stress responses (note that the LC is not directly involved in the PLR but indirectly influences it by modulating arousal and stress levels, which can affect the parasympathetic nervous system’s control of the pupil). The LC regulates the overall level of arousal in the brain; when arousal levels are high (e.g., during stress responses), the LC is more active, releasing norepinephrine [47]. This can increase the sensitivity of PLR, leading to altered pupil constriction [34]. The LC is also involved in attentional processes. When attention is focused on a specific stimulus, the LC can help to enhance the processing of that stimulus, including the PLR [48]. Overall, PLR is a complex neurological response that is mediated by the ANS and is influenced by both autonomic and cognitive factors. As a biomarker, PLR offers valuable insights into the integrity of the ANS and cognitive function.

### 4.2. Influence of the Menstrual Cycle on Parameters of the Autonomic Nervous System

By assessing HRV, BRS, MSNA, and PLR throughout the menstrual cycle, researchers can gain valuable insights into how the ANS is dynamically influenced by hormonal fluctuations. HRV can demonstrate the interaction between sympathetic and parasympathetic activity, which can reveal shifts in autonomic balance across the cycle, potentially explaining variations in mood, stress responses, and overall well-being. BRS, by assessing the body’s ability to regulate BP, and MSNA, by understanding the sympathetic activity, can provide insights into how hormonal changes impact cardiovascular control, which is crucial for overall health. Finally, PLR primarily assesses the non-cardiovascular ANS changes during different phases of the menstrual cycle, potentially influencing arousal, alertness, and responses to stress. By observing changes in these measures across the menstrual cycle, researchers can begin to understand how these ANS fluctuations contribute to the diverse physiological and psychological outcomes experienced by women, such as variations in mood, cognitive function, and even physical performance.

#### 4.2.1. Heart Rate Variability

HRV is a non-invasive and powerful tool that can be used to examine the influence of the menstrual cycle on the ANS. Previous research has found results that demonstrate the utility of monitoring the menstrual cycle. A recent meta-analysis of 37 studies and 1004 naturally menstruating women suggested a significant decrease in cardiac vagal activity from the follicular to the luteal phase in naturally menstruating biological women [49,50]. This suggests a potential shift towards increased SNS dominance, which may have implications for various physiological and psychological processes, such as mood, stress responses, and cardiovascular function. However, there are two studies that have reported otherwise. Blake et al. (2023) reported increased vmHRV during the luteal phase in 22 healthy biological women, while Brar et al. (2015) assessed HRV in 50 young healthy biological women with regular menstrual cycles, finding significantly higher mean HR and frequency-domain parameters, i.e., LF and HF, during the menstrual phase compared to the follicular phase [51,52]. Interestingly, in the latter study, the HRV was significantly higher in both time-domain (mean HR, RMSSD, and NN50) and frequency-domain parameters (VLF, LF, and HF) during the follicular phase when compared to the luteal phase, while the mean HR and the normalised LF were significantly higher in the luteal phase compared to the menstrual phase. These findings underscore the importance of considering the menstrual cycle when interpreting HRV data and have significant implications for understanding the physiological and psychological impact of hormonal fluctuations on women’s health.

Furthermore, Schmalenberger et al. (2020) performed an observational study on 31 biological women to determine the relationship between HRV and emotional affect during different phases of the menstrual cycle [49]. The results showed a decrease in vmHRV during the ovulatory-to-mid-luteal phase; however, it was not associated with premenstrual emotional changes. Interestingly, the subgroup of biological women whose vmHRV increased during the luteal phase had a significantly worsened negative affect pre-menstrually, which improved post-menstrually. It was proposed that an increase in the vmHRV could be a compensatory response in biological women with a significantly higher negative affect. Interestingly, a different study reported reduced vmHRV during the luteal phase, but only in women with more severe cases of PMS [6]. Another study looked into the association between vmHRV and progesterone during different phases of the menstrual cycle [53], finding a positive association between progesterone and vmHRV in the early follicular phase but not the periovulatory phase [53]. They also found a significant reduction in the vmHRV from the follicular to the luteal phase. Another study calculated HRV at rest and during lower-body negative pressure application to simulate hypovolaemia in biological women and found significantly lower resting values of LF but higher resting values of HF in the follicular phase as compared to biological women in the luteal phase during hypovolaemia LF; HF changed only during the luteal phase [54]. These results therefore may suggest that changes in HRV, particularly vagal activity, may serve as a potential biomarker for identifying women who may be more susceptible to premenstrual emotional disturbances and could inform personalised strategies for managing these symptoms.

Several studies have tried to assess the relationship between hormonal changes and HRV during the phases of the menstrual cycle. In one interesting study, postmenopausal women demonstrated lower baseline LF and HF measures compared to premenopausal women during the low oestrogen phase [55]. The HRV and cortisol levels during the menstrual cycle were studied in biological female athletes; no difference was noted in the cortisol levels between the phases of the menstrual cycle, while the time-domain parameters of HRV, including SDNN and RMSSD, were significantly higher in the menstruation phase [56]. A different study explored the relationship between oestrogen, progesterone, and HRV, and only progesterone showed a negative correlation with HRV [49].

#### 4.2.2. Baroreflex Sensitivity

To investigate the relationship between hormonal fluctuations and the BRS, several studies have examined these factors in biological women. One study evaluated cardiovagal BRS and circulating concentrations of oestrogen and progesterone in naturally menstruating women, as well as women on oral contraceptive pills (OCPs), during the early follicular/placebo pill and late follicular to early luteal/active pill phases of the menstrual cycle. In the early follicular phase or during the placebo pill phase, naturally menstruating women exhibited a lower BRS compared to biological women using OCPs. Furthermore, higher oestrogen concentrations, but not progesterone, were associated with predicting a lower BRS in these phases. During the late follicular to early luteal/active pill phase, higher progesterone concentrations were predictive of a lower BRS [57]. Another study assessed the autonomic profile in women with and without PMS and found a lower BRS in the late luteal phase in women with PMS compared to women without PMS [9]. One study found significant correlations between plasma oestrogen levels and BRS, as assessed by BP fluctuations induced by phenylephrine and Valsalva, in healthy women with regular menstrual cycles [58]. These findings highlight the complex relationship between hormonal fluctuations and BRS, suggesting that variations in oestrogen and progesterone levels across the menstrual cycle significantly influence autonomic regulation.

#### 4.2.3. Muscle Sympathetic Nerve Activity

There are a few studies that have assessed MSNA in biological women during different phases of the menstrual cycle; again, the results are contradictory. In one study, MSNA was measured at rest and during BP changes induced by sodium nitroprusside and phenylephrine infusions in nine healthy women with a regular menstrual cycle. MSNA was found to be significantly higher during the early follicular phase compared to the mid-luteal phase of the menstrual cycle [59]. Another study measured MSNA at rest and during mental stress simulated by mental arithmetic in 11 healthy women and found no difference in MSNA at rest or during the mental stress between the early follicular and mid-luteal phases [60]. The studies again demonstrate the complicated relationship between hormonal fluctuations and sympathetic activity in biological women [32].

#### 4.2.4. Pupillometry and Psychological Determinants of the Menstrual Cycle

Pupillometry can be used as a non-invasive tool for assessing ANS activity and cognitive function. While studies have explored the link between ANS function and cognitive performance during the menstrual cycle [61], few studies have examined the specific relationship between pupillary responses and this cyclic process. However, it is possible to speculate how pupil responses would be affected. Given the known hormonal fluctuations associated with the menstrual cycle, investigating its influence on pupillary responses could provide valuable insights into the relationship between ANS activity, cognition, and hormonal changes. During the follicular phase of the menstrual cycle, oestrogen levels rise, leading to increased parasympathetic activity [54]. This increased parasympathetic tone is reflected in smaller pupil sizes [48], which are potentially associated with a relaxed state [62]. Additionally, during the luteal phase, progesterone levels rise, leading to an increased sympathetic activity [63]. Heightened sympathetic arousal is associated with larger pupil sizes [64], suggesting a more alert or vigilant state [65]. More research is needed as results are inconsistent, yet there appears to be some evidence that pupillometry measures may vary over the course of the menstrual cycle and this variability may be associated with cognition.

The relationship between pupillary responses and cognitive function may be mediated by the ANS [66]. During periods of increased sympathetic arousal, larger pupil sizes are to be expected and individuals may be more focused and alert, which would enhance cognitive performance [67]. However, excessive sympathetic activity can also lead to stress and anxiety, which can impair cognitive function [68]. Therefore, further research into the relationship between the ANS, pupillary responses, and cognitive function is important for understanding the mechanisms underlying cognitive performance.

Overall, pupillometry appears to offer a promising approach for investigating the association between hormonal fluctuations, ANS activity, and cognitive function during the menstrual cycle. However, additional research is required.

## 5. The ANS, Cognition, and Emotional Valence During the Menstrual Cycle

The menstrual cycle, as well as fluctuations in the hormones oestrogen and progesterone during different phases of the menstrual cycle, has significant effects on the parameters of the ANS. However, whether and how the ANS affects cognition and emotions during the menstrual cycle is still unclear. An assessment of the ANS performed by various studies has shown varied results. This variability could be attributed to fluctuating hormonal concentrations, irregularity in the menstrual cycle itself, the varied length of the phases of the cycles, differences in the hormone levels based on the ages of these women, and genetic variabilities. However, most studies do show a propensity towards reduced vagal modulation during the luteal phase of the menstrual cycle in most biological women. The vagal modulation of cardiac activity is a well-established biomarker of psychopathology. An increase in the cardio-vagal activity has been shown to have an association with positive affect, and a decrease is associated with stress, while recovery from stress increases the vagal modulation [28,69]. Cardio-vagal modulation is often assessed by the HF component of the HRV. HF has been shown to have a positive correlation with empathy [70], social interaction [71], attention [72], behavioural regulation [68], executive function [68], positive empathic responsiveness, and attachment style [73]. Hence, it can be speculated that a reduction in the HF component of HRV during the luteal phase or even the later follicular phase can be associated with reduced empathy, difficulty in regulating behavioural responses, or maintaining appropriate social interactions; it can even be associated with difficulty maintaining attention or even the execution of social functioning. Severe cases of PMS or PMDD could very well have reduced cardio-vagal modulation. The association between low oestrogen levels and the reduced vagal modulation of the heart opens opportunities for the pharmacological management of PMS or even PMDD via hormonal replacements or hormone/oestrogen analogues. While these findings suggest a potential link between reduced vagal activity and the cognitive and emotional symptoms experienced by some women during the menstrual cycle, further research is required to determine the precise mechanisms underlying these associations and their clinical implications. Table 2 synthesises how the ANS, cognition, and emotional valence are affected across the menstrual cycle.

## 6. Limitations

There are several limitations that one needs to consider while investigating functions of the ANS in biological women during different phases of the menstrual cycle. Given the fact that both oestrogen and progesterone have a strong influence on the ANS and its parameters, fluctuations in these hormones could modify the results significantly. Irregular menstrual cycles can change the length of the different phases, thus changing the levels of circulating oestrogen or progesterone, making the ANS parameters incomparable. However, this situation can be tackled by simultaneously measuring the oestrogen or progesterone levels and the ANS parameters for comparison. Aside from this, the ANS parameters obtained from women on oral contraceptives or other hormonal preparations need to be interpreted with caution, as this will also modify the ANS parameters.

## 7. Conclusions

The menstrual cycle plays a major role in influencing emotional valence, cognition, and functions of the ANS in biological women. The ANS is integrated with cognition and emotion. Mental activity modulates ANS either by the direct regulation of the SNS and the PNS or indirectly through changes in respiration, posture, or by enacting behavioural decisions. This review has explored the multifaceted impact of the menstrual cycle on the ANS in biological women, which is a critical area of investigation given the significant influence of the ANS on various physiological and psychological processes. The ANS plays a fundamental role in regulating various homeostatic mechanisms of the human body, including cardiovascular regulation, neuroendocrine responses, pain perception, and cognitive and behavioural processes [1]. Its relationship with the brain involves the constant integration of sensory information from both internal and external environments, which allows for adaptive responses to maintain homeostasis [2].

An assessment of the ANS performed by various studies has shown varied results. This variability could be attributed to fluctuating hormonal concentrations, irregularity in the menstrual cycle itself, the varied length of the phases of the cycles, differences in the hormone levels based on the ages of these women, and genetic variabilities. However, most studies show a propensity towards reduced vagal modulation during the luteal phase of the menstrual cycle in most biological women. The vagal modulation of cardiac activity is a well-established biomarker of psychopathology. An increase in the cardio-vagal activity has been shown to have an association with positive affect, and a decrease is associated with stress, while recovery from stress increases the vagal modulation [28,69]. Cardio-vagal modulation is often assessed by the HF component of the HRV. HF has been shown to have a positive correlation with empathy [70], social interaction [71], attention [72], behavioural regulation [68], executive function [68], positive empathic responsiveness, and attachment style [73]. These cognitive and behavioural functions are intricately linked to the ANS and play a fundamental role in regulating various aspects of human behaviour, including cognition and emotion.

**Figure 2:**
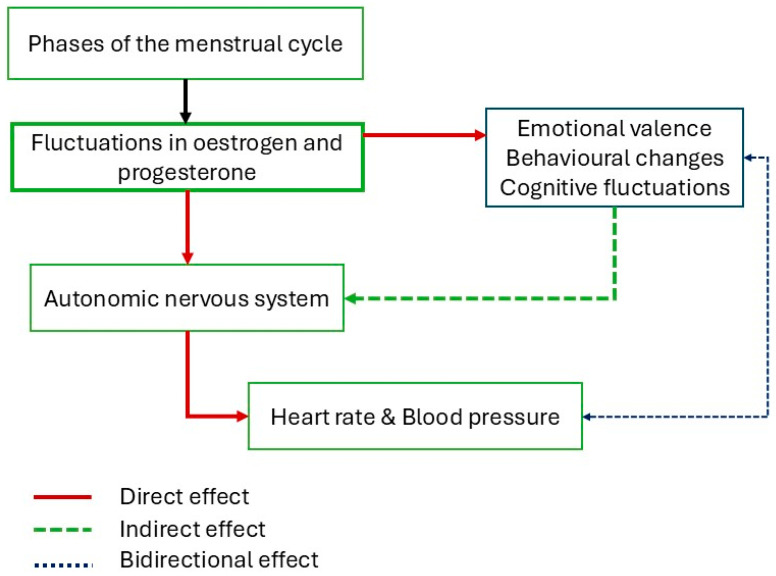
Flowchart depiction the direct, indirect, and bidirectional relationship between menstrual cycle phases, hormones, ANS, emotional valence, and cognition. Hormonal fluctuations during different phases of the menstrual cycle modify the ANS by either directly regulating the ANS centres in the brain or indirectly by modifying emotional valence and cognitive functioning.

Hence, it can be speculated that a reduction in the HF component of HRV during the luteal phase or even the later follicular phase, can be associated with reduced empathy, difficulty in regulating behavioural responses, or maintaining appropriate social interactions; it can even be associated with difficulty maintaining attention or even with the execution of social functioning. These findings therefore directly provide insights into the physiological underpinnings of conditions like PMS and PMDD. The observed association between reduced vagal activity and potential cognitive and emotional difficulties aligns with the known characteristics of these conditions, such as mood swings, anxiety, and cognitive difficulties, which are often linked to ANS dysregulation [9]. As demonstrated in Figure 2, the relationship between hormonal fluctuations, ANS activity, and cognitive and emotional states is evident across the different phases of the menstrual cycle. This figure visually represents the proposed model, illustrating how ovarian hormones modulate the ANS and subsequently impact cognitive and emotional processing.

Furthermore, the observed fluctuations in ANS function across the menstrual cycle have significant implications in relation to informing personalised healthcare approaches for women. By recognising the cyclical nature of ANS function, clinicians can tailor interventions, such as exercise routines or medication schedules, to optimise women’s health and well-being across the menstrual cycle. For example, interventions aimed at enhancing vagal tone, such as mindfulness or deep breathing exercises, could potentially be particularly beneficial during phases of the menstrual cycle that are associated with reduced vagal activity.

Finally, this research enhances our understanding of women’s performance in various domains. The potential impact of menstrual cycle-related ANS fluctuations on cognitive and emotional function, as suggested by the observed changes in HRV, has significant implications for women’s performance in various aspects of life, including work, academia, and social interactions. To further understand this area, future research would benefit from longitudinal designs with repeated measures in order to precisely track cognitive and mood changes alongside hormonal and ANS fluctuations during the menstrual cycle. There is a need for studies that use objective neuroimaging techniques to investigate the neural pathways and brain regions affected by these changes. This may contribute to the understanding of the direct causal links between hormonal fluctuations, ANS function, and cognitive and mood changes. Additionally, research should focus on interventions, such as mindfulness or cognitive behavioural therapy, to determine their efficacy in modulating ANS activity and mitigating cognitive and emotional symptoms throughout the menstrual cycle.

In conclusion, this review demonstrates that the menstrual cycle exerts a significant influence on the ANS, with significant implications for women’s health and well-being. By employing a combination of HRV, BRS, PLR, and other relevant assessments, we can further explore these intricate relationships and develop personalised healthcare approaches that address the unique physiological and psychological needs of women throughout their reproductive lifespan. There has been some research in understanding this complex relationship; however, there remains huge scope to perform further research in this area, including in relation to diagnostics and therapeutic interventions to combat the complex neurological and neuropsychological aspects of the menstrual cycle.

## Figures and Tables

**Table 1:** Summary of the key hormonal changes and physiological and psychological characteristics at each stage of the menstrual cycle.

Menstrual Stage	Key Hormonal Changes	Physiological and Psychological Characteristics
Menstruation and early follicular phase	Low oestrogen; low progesterone.	This phase is characterised by higher sympathetic activity and is associated with a lower baroreflex sensitivity (BRS), higher mean heart rate (HR), and higher low-frequency (LF) components of heart rate variability (HRV) compared to the late follicular phase. This phase may involve lower mood and increased muscle sympathetic nerve activity (MSNA). Reduced vagal modulation is common.
Follicular Phase	Oestrogen production gradually increases, peaking before ovulation. Some progesterone is also produced. Surge in luteinising hormone (LH) and follicle-stimulating hormone (FSH) at the end of this phase.	As oestrogen rises, there is an association with increased parasympathetic activity, but also reports of reduced vagal modulation. Studies show mixed results for HRV measures in this phase. MSNA can be higher early in this phase compared to the luteal phase, and a lower BRS is seen during its early part. Oestrogen’s impact on memory tasks has been observed, and it interacts with the cholinergic system. The LH surge, occurring just before ovulation, is linked to elevated pupillary light reflex (PLR) activity, suggesting a more active autonomic response.
Ovulation	Peak oestrogen, surge in luteinising hormone (LH) and follicle-stimulating hormone (FSH).	This is the release of a mature egg from the ovary, triggered by the LH and FSH surge. It is associated with high oestrogen levels. Additionally, an elevated PLR activity has been linked to the high LH levels, indicating a more active autonomic response during this phase.
Luteal Phase	Gradual increase in progesterone levels and an initial drop in the oestrogen levels followed by a gradual rise.	Characterised by reduced parasympathetic activity. Increased cardio-vagal activity is associated with positive affect, while decreased activity is linked to stress. A lower BRS is observed, especially in the late luteal phase for women with premenstrual syndrome (PMS). Some studies indicate higher vagally mediated heart rate variability (vmHRV), while others report reduced vmHRV in women with more severe cases of PMS. The rise in progesterone is linked to increased sympathetic activity and larger pupil sizes.

**Table 2:** Summary table for the current understanding of how each topic area is affected by the menstrual cycle.

Topic	Finding	Source
Emotional Valence	Phases with low oestrogen levels are associated with low mood in healthy biological women and depression in PMDD patients. Oestrogen affects dopamine metabolism, potentially reducing depression, and has anti-inflammatory effects related to depression.	[11,12,13]
Cognitive Functioning	Cognitive function is influenced by the menstrual cycle, with both progesterone and oestrogen affecting cognition. Oestrogen impacts memory tasks and interacts with the cholinergic system, while progesterone has neuroprotective effects. Inconsistent findings exist.	[14,15,16,17,18,19]
Heart Rate Variability (HRV)	A meta-analysis suggested a significant decrease in cardiac vagal activity from the follicular to the luteal phase. Other studies show increased vmHRV during the luteal phase; a higher mean HR, LF, and HF during menstruation compared to the follicular phase; and reduced vmHRV during the luteal phase in women with more severe cases of PMS.	[6,49,50,51,53,54,55,56]
Baroreflex Sensitivity (BRS)	BRS was lower in naturally menstruating women during the early follicular/placebo pill phases compared to women on OCP, and higher oestrogen (but not progesterone) predicted a lower BRS. Higher progesterone predicted a lower BRS during the late follicular to early luteal/active pill phase. A lower BRS was found in the late luteal phase in women with PMS.	[9,57,58]
Muscle Sympathetic Nerve Activity (MSNA)	Some studies found significantly higher MSNA during the early follicular phase compared to the mid-luteal phase. Other studies found no difference in MSNA at rest or during mental stress between the early follicular and mid-luteal phases.	[32,59,60]
Pupillary Light Reflex (PLR)	Oestrogen rise in the follicular phase is linked to increased parasympathetic activity and smaller pupil sizes. Progesterone rise in the luteal phase is linked to increased sympathetic activity and larger pupil sizes. More research is needed due to inconsistent results.	[48,54,62,63,64,65,66,67,68]
ANS, Cognition, and Emotional Valence	Reduced vagal modulation is often observed during the luteal phase. Increased cardio-vagal activity is associated with positive affect, while a decrease is linked to stress. The HF component of HRV (a measure of cardio-vagal modulation) correlates positively with empathy, social interaction, attention, and executive function.	[28,68,69,70,71,72,73]

## Data Availability

No new data were created or analyzed in this study. Data sharing is not applicable to this article.
